# Enhanced Cell-Based Detection of Parvovirus B19V Infectious Units According to Cell Cycle Status

**DOI:** 10.3390/v12121467

**Published:** 2020-12-18

**Authors:** Céline Ducloux, Bruno You, Amandine Langelé, Olivier Goupille, Emmanuel Payen, Stany Chrétien, Zahra Kadri

**Affiliations:** 1 Laboratoire Français du Fractionnement et des Biotechnologies (LFB), 3 Avenue des Tropiques, BP 305, Courtabœuf CEDEX, 91958 Les Ulis, France; celine.ducloux@gmail.com (C.D.); you@lfb.fr (B.Y.); angeleamandine@lfb.fr (A.L.); 2 Division of Innovative Therapies, UMR-1184, IMVA-HB and IDMIT Center, CEA, INSERM and Paris-Saclay University, F-92265 Fontenay-aux-Roses, France; olivier.goupille@cea.fr (O.G.); emmanuel.payen@cea.fr (E.P.); stany.chretien@cea.fr (S.C.)

**Keywords:** B19 parvovirus, detection, erythroid cells, cell cycle, permissivity

## Abstract

Human parvovirus B19 (B19V) causes various human diseases, ranging from childhood benign infection to arthropathies, severe anemia and fetal hydrops, depending on the health state and hematological status of the patient. To counteract B19V blood-borne contamination, evaluation of B19 DNA in plasma pools and viral inactivation/removal steps are performed, but nucleic acid testing does not correctly reflect B19V infectivity. There is currently no appropriate cellular model for detection of infectious units of B19V. We describe here an improved cell-based method for detecting B19V infectious units by evaluating its host transcription. We evaluated the ability of various cell lines to support B19V infection. Of all tested, UT7/Epo cell line, UT7/Epo-STI, showed the greatest sensitivity to B19 infection combined with ease of performance. We generated stable clones by limiting dilution on the UT7/Epo-STI cell line with graduated permissiveness for B19V and demonstrated a direct correlation between infectivity and S/G2/M cell cycle stage. Two of the clones tested, B12 and E2, reached sensitivity levels higher than those of UT7/Epo-S1 and CD36^+^ erythroid progenitor cells. These findings highlight the importance of cell cycle status for sensitivity to B19V, and we propose a promising new straightforward cell-based method for quantifying B19V infectious units.

## 1. Introduction

Human Parvovirus B19 (B19V), a member of the genus *Erythroparvovirus* of the Parvoviridae family, is a widespread virus that is pathogenic to humans [1]. The genome of B19V is a linear 5.6-kb single-stranded DNA, packaged into a 23–28 nm non-enveloped icosahedral capsid [2]. Replication occurs in the nucleus of infected cells, via a double-stranded replicative intermediate and a rolling hairpin mechanism. B19V infection has been associated with a wide spectrum of diseases, ranging from erythema infectiosum during childhood (known as the “fifth disease” and characterized by a common “slapped-cheek” rash) [3,4], to arthropathies [5], severe anemia [6] and systemic manifestations involving the central nervous system, heart and liver, depending on the immune competence of the host [7]. Productive B19V is restricted to human erythroid progenitor cells [8], and its clinical manifestations are linked to the destruction of infected cells [9]. Indeed acute B19V infection can cause pure red-cell aplasia in patients with pre-existing hematologic disorders leading to high levels of erythrocyte turnover (e.g., in sickle cell disease or thalassemia patients) [10,11,12], and in immunocompromised or transplanted patients [13]. The virus is transmitted via respiratory secretions and feto-maternal blood transfers. During pregnancy, infection with B19V can cause non-immune fetal hydrops, congenital anemia, myocarditis and terminal heart failure, leading to spontaneous abortion or stillbirth of the fetus [14,15]. The high prevalence of B19V infection in the general population and the large number of blood donations used in the manufacture of plasma-derived factor concentrates favors high levels of contamination. Few reports [16,17,18] of clinical B19V infection resulting from the transfusion of contaminated blood components or infusions of plasma-derived medicinal products suggests that measures to reduce the transmission risk (e.g., nucleic acid testing (NAT) and/or virus removal/inactivation steps) are effective. This efficacy has led to NAT becoming the gold standard for testing products of biological origin [19]. Indeed, to counteract B19V blood-borne patient’s contamination, evaluation of B19 DNA in plasma pools and viral inactivation/removal steps are performed, but nucleic acid testing did not correctly reflect B19V infectivity [17,20].

Reducing the risk of B19V infection is mandatory for suppliers of blood-derived products worldwide [21]. The elimination of viruses must be assessed in processes for the production of plasma-derived medical products, but B19V DNA quantification may be inadequate: viral DNA can persist in the serum for months after acute infection, and its levels are therefore not necessarily correlated with infectivity [20,22]. The use of titration-based B19V infectivity assays is therefore essential. Moreover, the last few decades have seen the development of regenerative therapies based on Hematopoietic or Mesenchymal Stem Cells (HSC or MSC) from bone marrow and synovium donors, respectively. According to the guidelines ensuring clinical grade of human stem cells, one of the major safety concerns is detecting latent viruses in cell sources [13,23]. Stem cells seem to act as a latent reservoir for B19 infection [24]. If viral contamination is overlooked at initial screening, then the virus may be amplified during culture before transplantation, through the reactivation of latent B19V [25]. For all these reasons, a practical and sensitive in vitro method for assessing B19V infectivity is required. However, efforts to develop such methods have been hampered by the lack of suitable B19-sensitive cell lines.

B19V displays a marked tropism for erythroid progenitor cells (EPC), but there is still no well-established cell line for B19V infection. The UT7/Epo-S1 cell line [26], an erythropoietin (Epo)-dependent subclone derived from the mega-karyoblastoid cell line UT-7 [27], is the most widely used cell model, because of its high sensitivity to B19V replication and transcription [28]. However, B19V infection is limited to a small number of cells (1%–9%, versus 30%–40%% for primary or immortalized erythroid progenitor cells) [29,30,31].

Here, we compared the sensitivities of a number of different erythroid cell lines to B19 infection with that of UT7/Epo-S1. We generated stable clones with graduated permissivity to B19V from a single parental cell line. Using the FUCCI (fluorescent ubiquitination cell cycle indicator) system to analyze cell cycle, we demonstrated a direct correlation between infectivity and S/G2/M cell cycle stage, and characterized two clones, B12 and E2, with sensitivity to B19V up to 35 time higher than that of UT7/Epo-S1. 

## 2. Materials and Methods 

### 2.1. Cell Lines

Three distinct UT-7/Epo cell lines were used: (1) UT7/Epo-S1, a clone of UT7/Epo [32], was obtained from Dr Kazuo Sugamura (Tohoku University Graduate School of Medicine, Japan). (2) UT7/Epo-APHP and UT7/Epo-Cl3, a subclone isolated from UT7/Epo-APHP, were a gift from Dr Morinet (APHP: Assistance Publique - Hôpitaux de Paris, Saint Louis Hospital). (3) UT7/Epo-STI cells were derived from UT-7/GM cell line and were maintained at low passage, with stringency for erythroid features [33]. UT7 cell lines were maintained at 37 °C, under an atmosphere containing 5% CO_2_, in alpha minimum essential medium (αMEM) supplemented with 10% fetal calf serum (FCS), 2 mM L-glutamine (Hyclone), 100 U/mL penicillin, 100 µg/mL streptomycin and 2 U/mL recombinant human (rh) Erythropoietin (rh-Epo, Euromedex, RC213-15). Where specified, 0.5 µM JQ1 (Sigma-Aldrich, France) or 2 ng/mL TGF-β (Peprotech, France) was added to the culture medium for two days before B19V infection. TF1 and TF1-ER erythroleukemia cells [34] were maintained in Roswell Park Memorial Institute (RPMI) 1640 medium supplemented with 10% FCS, 2 mM L-glutamine (Hyclone), 100 U/mL penicillin, 100 µg/mL streptomycin and 2 U/mL rh-Epo or 25 ng/mL human granulocyte macrophage colony-stimulating factor (GM-CSF, Peprotech). KU812Ep6 cells [35], a gift from Dr Sakai (Health Science Research Resources Bank, Tokyo, Japan), were maintained in RPMI-1640, 2 U/mL rh-Epo, 10% FCS, 100 U/mL penicillin, 100 µg/mL streptomycin and Insulin Transferrin Selenium-X supplement (ITS-X, Gibco), at 37 °C, 5% CO_2_. Human embryonic kidney (HEK) 293T and NIH-3T3 cells were maintained in Dulbecco’s modified Eagle’s medium (DMEM) supplemented with 10% FCS, 2 mM L-glutamine, 100 U/mL penicillin and 100 µg/mL streptomycin.

### 2.2. CD36^+^ Erythroid Progenitor Cell (EPC) Line Generation

Umbilical cord blood (CB) units from normal full-term deliveries were obtained, with the informed consent of the mothers, from the Obstetrics Unit of Saint Louis Hospital, Paris, and collected in placental blood collection bags (Maco Pharma, Tourcoing, France). Blood mononuclear cells were purified by Ficoll density gradient separation (Leucosep, Greiner Bio-one) and Hanks medium (Thermo-Fisher). Low-density cells were recovered and enriched for CD34^+^ cells by automated cell sorting (CD34 isolation kit and autoMACS System, Miltenyi Biotec). CD34^+^ cells were cultured in serum-free expansion medium: Iscove’s Modified Dulbecco’s Medium (IMDM), 15% BIT 9500 (BSA-Insulin-Transferrin, Stem Cell Technologies), 60 ng/mL rh-Stem Cell Factor (SCF), 10 ng/mL rh- interleukin-3 (IL-3), 10 ng/mL rh-IL-6, 2 U/mL rh-Epo, 100 U/mL penicillin and 100 μg/mL streptomycin. After seven days of culture, CD36^+^ cells were isolated with biotin-coupled anti-CD36 antibody and anti-biotin microbeads on an autoMACS System. CD36^+^ EPCs were obtained by lentivirus-mediated transduction with the *hTERT* and *E6/E7* genes from human papillomavirus type 16, as previously described [36], and were grown in expansion medium to generate a continuous CD36^+^ EPC line.

### 2.3. B19 Virus Stock and Cell Inoculation

Plasma samples containing high titers of infectious B19V from asymptomatic donors at blood donation were provided by the Etablissement Français du Sang (EFS). Determination of anti-B19V IgG and IgM in plasma samples was performed using respectively LIAISON^®^ Biotrin Parvovirus B19 IgG (cat. N° 317000) and IgM (cat. N° 317010) (Diasorin S.A., Antony, France). According to manufacturer’s instructions, samples were analyzed by Chemiluminescence ImmunoAssay (CLIA) and obtained results were compared to provided negative and positive controls. Plasma samples were determined to be qualitatively negative for both B19V IgG and IgM, with a viral titer of 10^11^ B19V DNA genome equivalent (ge)/mL. The infection assay was performed using a protocol similar to that previously described [37]. Briefly, cells were maintained in exponential growth condition by dilution to 0.3 × 10^6^ cells/mL the day before infection. On the day of infection, cells were washed and diluted in FCS-free medium without Epo, at a density of 10^7^ cells/mL. B19V inoculation was carried out in a 96-well plate, with 10 µL of cell suspension (10^5^ cells) and 50 µL of a 100-fold dilution of B19V plasma (10^9^ ge/mL), corresponding to a mean of 500 ge/cell. The cells were then incubated at 4 °C for 2 h, and then at 37 °C, for 1 h, under an atmosphere containing 5% CO_2_. We added 140 µL of complete medium and maintained the cells in culture until 72 h. Where specified, we added chloroquine (CQ) to the complete medium, at a final concentration of 25 µM. Cell viability was assessed by Trypan blue exclusion test (0.4% in PBS, Thermo Fisher Scientific), by counting blue and total cells under a microscope, with a hemocytometer. After correction for the dilution factor, viability was calculated as follows: percent of viable cells = (1 − (number of blue cells/number of total cells)) × 100. At 24, 48 or 72 h post infection (hpi), cells were centrifuged (8 min at 300× *g*), supernatants were discarded and cell pellets were frozen at −80 °C until analysis.

### 2.4. Fucci2a Lentivirus Production and Cell Transduction 

The Fucci2a DNA sequence [38] (RDB13080, RIKEN BioSource Center) was synthetized into the LTGCPU7 lentiviral vector backbone [39] without the puromycin resistance-gene cassette, and under the control of the EF1α promoter and enhancer (GenScript). Lentiviral particles were produced by the transient transfection of HEK293T cells with the five-plasmid packaging system, by PEIpro (Polyplus transfection), as previously described [40]. These particles were then concentrated by ultracentrifugation. Infectious titers were determined in NIH-3T3 cells. We transduced 0.5 × 10^6^ UT7/Epo-STI cells with FUCCI particles at a mean of infection of 10 in 200 µL of complete medium, and the cells were kept at 37 °C for 4 h. Cells were subsequently diluted at 0.1 × 10^6^ cells/mL. On days 6 and 9 post-transduction, cells were analyzed by cytometry for the expression of FUCCI proteins.

### 2.5. UT7/Epo-FUCCI Clones Generation

UT7/Epo-FUCCI refers further to a UT7/Epo-STI pool expressing FUCCI. UT7/Epo-FUCCI clones were isolated in a U-bottom 96-well plate, by limiting dilution, with one seeded cell per well in 100 µL of complete medium. Cells were visualized by microscopy, and wells containing more than one cell or non-fluorescent cells were excluded. Clones were then separately expanded with an assigned name corresponding to their location on the plate. After expansion, each clone was considered further as a new cell line. A cell bank of 156 isolated clones was constituted (stored at 80 °C in 90% FCS, 10% DiMethyl SulfOxide (DMSO)). To reach exponential growth, cells were diluted at 0.3 × 10^6^ cells/mL. The day after dilution, isolated clones were subjected to FUCCI expression profiling. The stability of the cell cycle profiles of the isolated clones was controlled both sequentially, for at least five independent cultures, and for 10 passages of the same culture.

### 2.6. Flow Cytometry Analysis of Cell Cycle Status 

FUCCI-2a stable transduction in UT7-FUCCI cells allows the expression of two fluorescent proteins (m-Venus and m-Cherry) fused with specific cell cycle proteins (respectively Geminin and Cdt1). Fusion proteins are continuously expressed, but as Cdt-1 and Geminin are ubiquitinated and degraded by the proteasome at specific cell cycle stages, resting fluorescence reflects cell cycle status: during G_1_ phase, the geminin-mVenus fusion protein is degraded, while the Cdt1–mCherry fusion is expressed resulting in red-fluorescence. During G_1_/S transition, both proteins are present in the cells and the cell nuclei appear yellow as the green and red fluorescence overlay. In the S, G_2_, and M phases, the Cdt1–m-Cherry fusion is degraded, leaving only the geminin–m-Venus fusion and resulting in a green-fluorescence signal. This dynamic color change from red to yellow to green serves as an indicator of the progression through cell cycle and division. Fucci2a bicistronic expression was monitored with an LSRFortessa cytometer (BD Biosciences, Le Pont de Claix, France). Fluorescent fusion proteins were detected with the 488 nm blue laser and a 530/30 nm bandpass filter (B530/30) for mVenus-hGeminin, and the 590 nm yellow laser and a 610/20 nm bandpass filter (Y610/20) for mCherry-hCdt1. For alternative monitoring of the cell cycle according to DNA content, cells were stained with the permeable DNA dye Hoechst 3342 (10 μg/mL) for 1 h at 37 °C, and immediately analyzed for DNA content with the 355 nm violet laser and a 450/40 nm bandpass filter (V450/40). FACSDiVa and FlowJo X software (BD Biosciences, Le Pont de Claix, France) were used to operate the instrument and for data analysis, respectively.

### 2.7. RNA Extraction and Duplex RT-qPCR

**Figure 1:**
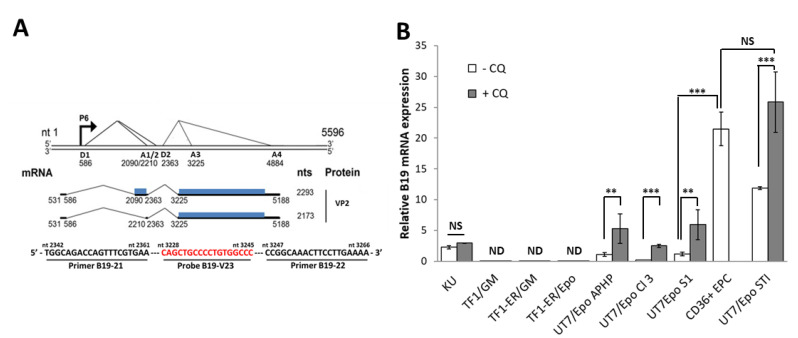
Comparison of the B19V sensitivity and permissiveness of hematopoietic cell lines. (**A**) B19V transcription profile (adapted from Ganaie et al., J Virol. 2018 [7]). The major transcription unit of the B19V duplex genome (GenBank accession no. AY386330) is shown to scale at the top, with the P6 promoter, 2 splice donors (D1, D2) and 4 acceptors (A1 to A4) sites. In blue, mRNA encoding the VP2 viral proteins, with nucleotides (nts). At the bottom, the primers and probe used for the RT-PCR amplification of VP2. (**B**) Bone marrow-derived primary Erythroid Progenitor Cells (CD36^+^ EPCs), human leukemic cell lines (TF1, TF1-ER, UT7/Epo-APHP, UT7/Epo-STI) and isolated clones (KU812Ep6, UT7/Epo-cl3 and UT7/Epo-S1) were seeded in triplicate and inoculated with or without B19V in culture medium supplemented with Epo (2 U/mL)(/Epo), or granulocyte macrophage colony-stimulating factor (GM-CSF) (25 ng/mL)(/GM) for TF1 and TF1-ER. When specified, cells were cultivated with (+) or without (-) Chloroquine (CQ, 25 µM). No CQ treatment was applied to CD36+EPC. 72 h post-infection, cells were pelleted and lysed. RNA was extracted and analyzed by RT-qPCR for VP2 to quantify B19 viral genome expression, and for β-actin for cell number normalization. For each cell line, the results without B19V correspond to the negative control. Relative B19V threshold cycle (Ct) values were normalized relative to b-actin Ct and expressed according to the 2^−^^ΔΔ^^Ct^ method with normalization against mean VP2 expression for UT7/Epo-S1 cells without CQ (*n* = 6). Results are presented as means + SEM of 3 independent experiments. ** *p* < 0.01; *** *p* < 0.001; NS = No Significance. ND = Not detected.

Total RNA was extracted from cell pellets with the RNeasy 96 QIAcubeHT kit and a QIAcubeHT machine, according to the manufacturer’s instructions. The extraction step included DNase treatment for 15 min, to decrease the risk of genomic DNA amplification during PCR. Real-time reverse transcription-quantitative PCR (RT-qPCR) was performed with the *Taq*man Fast Virus one-step PCR kit (Applied Biosystems). B19 VP2 transcripts were amplified with the sense primer B19-21 5′-TGGCAGACCAGTTTCGTGAA-3′ (nts 2342-2361), the antisense primer B19-22 5′-CCGGCAAACTTCCTTGAAAA-3′ (nts 3247-3266) and the probe B19-V23 5′-VIC-CAGCTGCCCCTGTGGCCC-3′ (nts 3228-3245). For control and normalization with respect to the number of cells, we used a duplex strategy. A target sequence of the spliced beta actin transcript was selected and amplified with the sense primer actin-S 5′-GGCACCCAGCACAATGAAG-3′, the antisense primer actin-AS 5′GCCGATCCACACGGAGTACT-3′ and the probe actin-FAM 5′-FAM-TCAAGATCATTGCTCCTCCTGAGCGC-3′. Reactions were performed on 5 µL of extracted RNA with the Quant Studio 3 PCR system. The reaction began with reverse transcription at 48 °C for 15 min, followed by inactivation of the reverse transcriptase and activation of the polymerase by heating at 95 °C for 10 min, followed by 40 cycles of 15 s at 95 °C and 30 s at 60 °C. The PCR program was optimized for amplification of the VP2 spliced transcripts rather than the VP2 genomic sequence (Figure 1A).

### 2.8. Statistical Analysis

Values are expressed as mean ± SEM. Data were determined to be normally distributed as the max and the min values in each data set were <3.sd from the mean. Data were analyzed using Prism 6.0 (GraphPad Software) by one-tailed Student’s t-test or one-way ANOVA with Bonferroni post-test (α < 0.05). Parameters measured over multiple time points were analyzed with two-way ANOVA with Bonferroni post-test and time was within subject factor. The significance level displayed on figures are as follows: * *p* <0.05, ** *p* <0.01, *** *p* <0.001 and “NS” means no significance. Samples and experiment sizes were determined empirically to achieve sufficient statistical power.

## 3. Results

To assess and compare the degree of permissivity to B19V, hematopoietic cell lines were infected with B19V and maintained for 72 h. Where specified, chloroquine (CQ) was added to boost virus entry and prevent the degradation of incoming viruses through a blockade of lysosome transfer [41]. Active transcription of the B19V genome in host cells was evaluated by RT-qPCR for the *VP2* capsid gene (Figure 1A), with normalization to beta-actin gene expression. As a reference to calculate relative B19 mRNA expression, the value for UT7/Epo-S1 without chloroquine was set to 1 (Figure 1B). As previously reported, UT7/Epo-S1 and KU812Ep6 cells were less permissive to B19V than CD36^+^ EPCs. Chloroquine treatment markedly enhanced UT7/Epo-S1 sensitivity (five-fold), but without reaching the level obtained for CD36^+^ EPCs. VP2 expression was undetectable in both the parental TF1 erythroleukemia cell line and a TF1-ER cell line expressing a full Epo-receptor, under the control of GM-CSF or Epo, with or without chloroquine treatment. Among all UT7/Epo cell lines tested, UT7/Epo-STI was the UT7/Epo cell line tested with the highest sensitivity to B19V, with B19 mRNA levels 11.8 ± 0.2 times higher than those in UT7/EpoS1. Sensitivity was enhanced by chloroquine treatment and reaches an equivalent level compared to CD36^+^ EPCs (UT7/Epo-STI + CQ: 25.8 ± 4.9 vs. CD36^+^ EPCs 21.49 ± 2.7).

**Figure 2:**
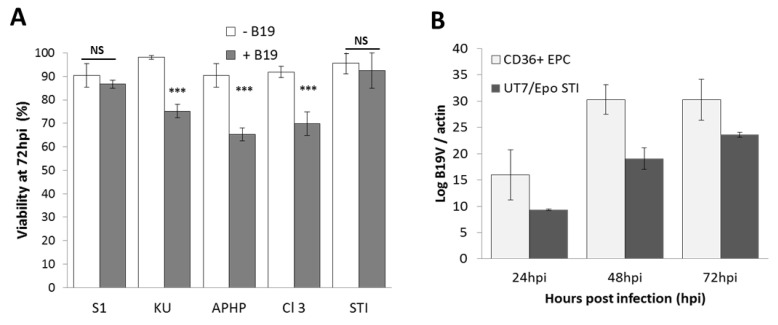
Comparison of B19V sensitivity of hematopoietic cell lines. (**A**) Cell viability was assessed 72 h post-infection. The results shown are the means + SD of three independent experiments. (**B**) UT7/Epo-STI cells and CD36^+^ EPCs were cultured in triplicate, with or without B19V, for 72 h. At 24, 48 and 72 h post-inoculation, cells were collected by centrifugation. RNA was extracted from the cell pellet and VP2 mRNA levels were analyzed to quantify B19 viral DNA expression, and β-actin mRNA levels were analyzed for cell number normalization. For each cell line, results without B19V correspond to the negative control. Relative B19V threshold cycle (Ct) values were normalized relatively to the β-actin (log B19V/actin). The results shown are the means + SEM of three independent experiments. *** *p* <0.001; NS = No Significance.

This increase in sensitivity was not due to resistance to B19V-induced cytotoxicity (Figure 2A). The expression kinetics of UT7/Epo-STI B19V were similar to those for CD36^+^ EPC, with a maximum reached at 72 h post-infection for both cell lines (Figure 2B).

**Figure 3:**
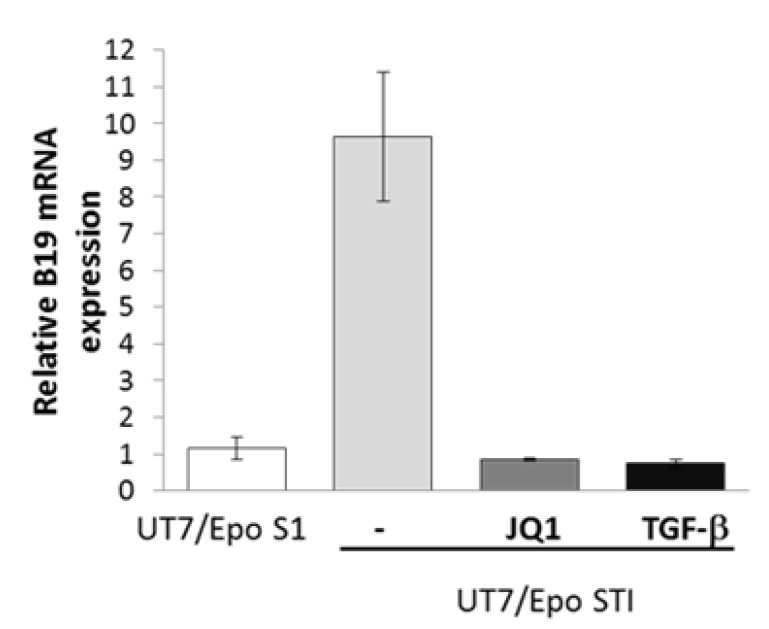
B19V-sensitivity of UT7/Epo-STI cells is linked to maturation stage. UT7/Epo-STI cells were cultured for 48 h before inoculation with B19V, without (-) or with JQ1 (0.5 µM) or TGF-β (2 ng/mL). 72 h post-inoculation, relative levels of B19V VP-2 mRNA were evaluated with UT7/Epo-S1 cells as the reference.

As sensitivity to B19V is directly linked to maturation stage, we therefore subjected UT7/Epo-STI cells to the chemical (JQ1) or hormonal (TGF-β) induction of erythroid differentiation two days before B19V infection. Both treatments decreased B19V infection by a factor of about 10, to levels similar to those obtained for UT7/Epo-S1 (Figure 3).

**Figure 4:**
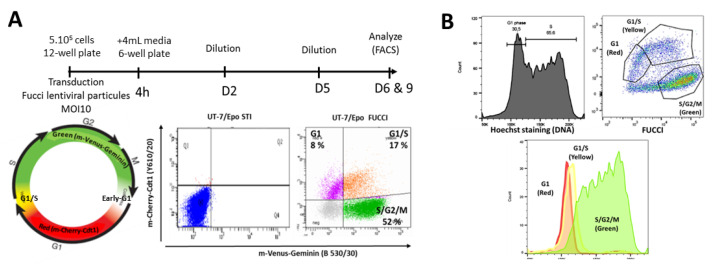
Generation of a UT7/Epo-STI cell line with stable expression of the Fluorescence Ubiquitination Cell Cycle Indicator (FUCCI). (**A**) Experimental design for the generation of the UT7/Epo-FUCCI cell line. Bottom: Two-color cell cycle mapping with the FUCCI2a Cell Cycle Sensor and right, flow cytometry analysis of exponentially growing UT7/Epo-STI and UT7/Epo-FUCCI cells. The profile shown corresponds to one representative experiment. (**B**) DNA content and FUCCI profiles for the same sample. Exponentially growing UT7/Epo-FUCCI cells were stained with Hoechst 33342. DNA content (Hoechst on the *x*-axis; cell count on the *y*-axis) and FUCCI proteins (m-Venus on the *x*-axis; m-Cherry on the *y*-axis) were concomitantly evaluated by flow cytometry. Bottom: Overlay of gated cell cycle populations, as determined by FUCCI analysis with DNA content profile.

We chose to select clones according to cell cycle status, to increase sensitivity to B19V. UT7/Epo-STI cells were transduced with FUCCI (Fluorescence Ubiquitination Cell Cycle Indicator) lentiviral particles to generate the UT7/Epo-FUCCI cell line (Figure 4A). The FUCCI cell cycle sensor allows cell cycle analysis of living cells. The UT7/Epo-FUCCI cell line presents three different color profiles, from green, corresponding to the S, G2 and M phases, to red, consequent to G1 phase, with a green plus red (yellow) overlay indicating the G1-to-S transition. We checked that these dynamic color changes correctly represented progression through the cell cycle and division, by staining the DNA content of UT7/Epo-FUCCI cells with Hoechst stain (Figure 4B). An overlay of the DNA staining and FUCCI profiles resulted in a perfect match between the cell cycle status assigned by the FUCCI technique and that assigned on the basis of DNA content: G1 (red) FUCCI cells were detected at a DNA content of 2N, whereas cells at S-G2-M (green) had DNA content peaks of 2N to 4N, consistent with the expected replication of the DNA replication before mitosis. The G1/S transition phase (yellow) population was located at the 2N peak, with a slight shift from G1 cells. Overall, these results confirm that FUCCI is an appropriate cell cycle indicator for UT7/Epo cells.

We then generated different UT7/Epo-FUCCI clones, each obtained by limiting dilution and culture from a single fluorescent cell. Unlike the UT7/Epo-FUCCI pool, these clones were generated from single cells and 100% of the cells were therefore transduced: the colorless cells of the FUCCI profile correspond to the early G1 (eG1) phase and were included in the G1 phase for the purposes of this analysis. We isolated 156 independent clones and expanded each as new sub-lines. FUCCI-negative clones, accounting for one third of the cells isolated, were excluded. We studied the cell cycle status of FUCCI-positive clones. We defined three types of cell cycle profile in a total of 97 clones: (1) 54 clones presented a cell cycle with more than 60% of the cells in G1 phase (55.7% of clones); (2) 29 clones presented a balanced distribution of cells between the G1 and S/G2/M phases (29.9% of clones); (3) 14 clones had a high percentage of cells in the S/G2/M phases (14.4% of clones). With the aim of analyzing these three types of cell cycle profile, we selected 11 isolated clones in regard to the diversity of their cell cycle patterns at exponential growth (Figure S1).

**Figure 5:**
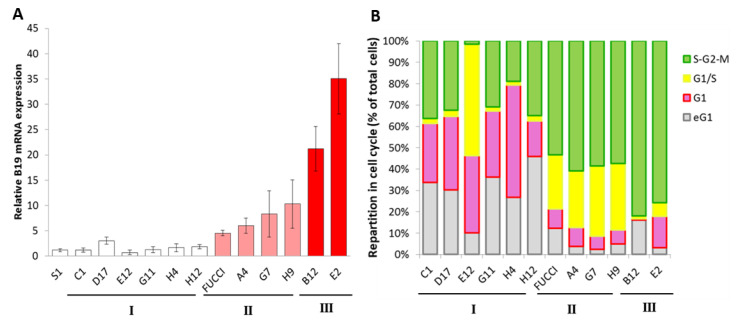
Improvement of B19V sensitivity and permissiveness according to cell cycle status. (**A**) UT7/Epo-S1 cells (S1), UT7/Epo-STI cells expressing the FUCCI system (FUCCI) and 11 UT7/FUCCI-derived isolated clones were inoculated with B19V. Relative levels of B19V mRNA were determined 72h post-infection, with UT7/Epo-S1 as the reference, and cell lines were classified on the basis of B19V sensitivity as group I for S1-equivalent clones, group II for FUCCI-equivalent clones, and group III for highly permissive clones. The results shown are the means + SD of 3 independent experiments for groups I and II, and *n* = 9 for group III clones. (**B**) The cell cycle status of exponentially growing FUCCI cell lines and isolated clones was assessed by flow cytometry. The results shown are the means + SEM of three independent measurements.

We evaluated sensitivity to B19V of exponentially growing selected clones as previously described (Figure 5A). Permissivity ranged from 1-fold to 35-fold relative to UT7/Epo-S1. Three populations were assigned: group I, with a sensitivity close to that of UT7/Epo-S1 (six clones); group II, gathering clones with UT7/Epo-FUCCI-like permissivity (three clones); group III, containing clones B12 and E2, displaying remarkable sensitivity to B19V infection. Interestingly, classification based on B19V sensitivity seemed to group together clones with similar cell cycle patterns (Figure 5B). The cell cycle profiles of group I clones displayed a predominance of the G1 phase. Group II clones displayed a balance between the G1 and S/G2/M phases, as observed for the original UT7/Epo-FUCCI pool. Finally, the S/G2/M cell population predominantly represents the group III profile, with 82% and 75.8% for B12 and E2, respectively.

**Figure 6:**
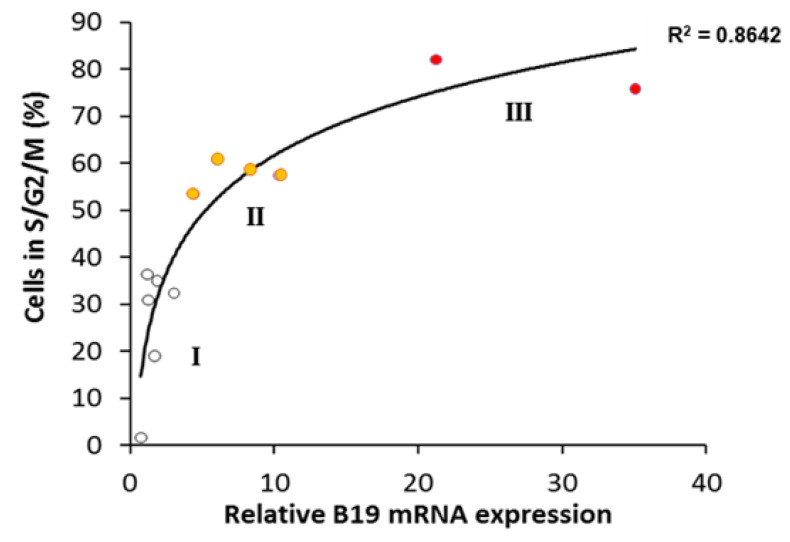
Comparison of relative levels of B19 mRNA (as in Figure 5A) and cell cycle status (as in Figure 5B). Each dot corresponds to the mean result for a single cell line or clone (*n* = 3), classified to groups I (white marks ○), II (orange marks ●) and III (red marks ●). Logarithmic regression analysis and R^2^ values are presented.

We evaluated the correlation between cell cycle stage and B19V sensitivity, by analyzing the correlation of the coefficient of determination (R^2^) obtained for eG1, G1, G1/S and S/G2/M with B19V mRNA levels (Figure S2 and Figure 6). For G1 cell cycle parameter, R^2^ was low, with values of 0.3743 for early G1 (eG1) and 0.5148 for G1.

The highest value was obtained for the S/G2/M phase of the cell cycle, with R^2^ = 0.8642, demonstrating an excellent agreement between the percentage of cells in S/G2/M stage and B19V sensitivity (Figure 6).

**Figure 7:**
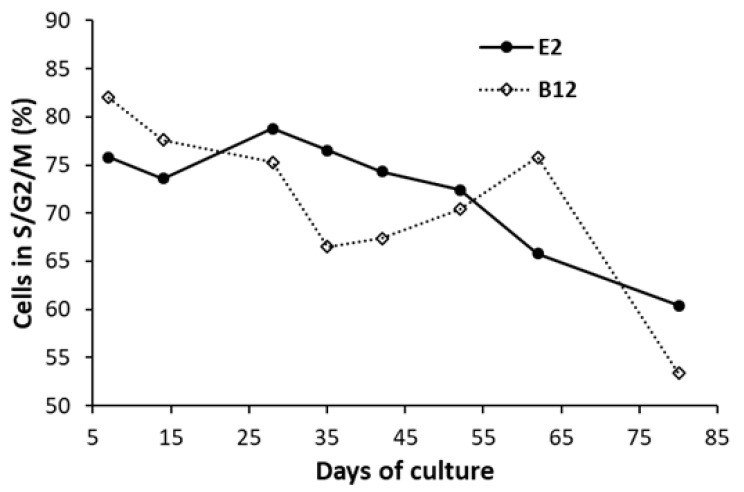
S/G2/M cell percentage of E2 and B12 clones during serial culture passage. UT7/Epo-FUCCI clone E2 and B12 were maintained in culture during 80 days after thawing with up to 25 culture passages. At indicated time points, cell cycle was evaluated by flow cytometry according to specific FUCCI protein expression. The graph represents the evolution of the percentage of cells in S/G2/M cell cycle status during cell culture, as calculated in Fig S3A (E2) and B (B12) Results shown are the means of three independent measurements.

Results obtained show that E2 and B12 are the most permissive clones. In order to assess the stability of their cell cycle profile, E2 and B12 were submitted to serial culture passage and cultivated for up to 80 days. Cell cycle profiles were analyzed by flow cytometry according to FUCCI expression (Figure 7 and Figure S3).

**Figure 8:**
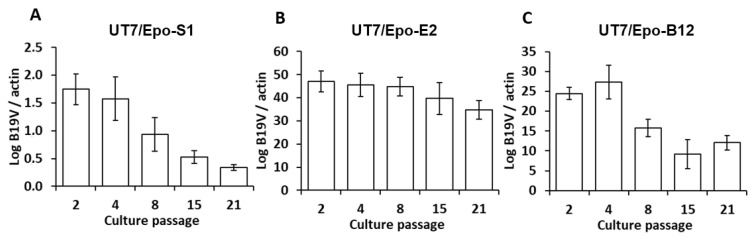
B19V permissivity throughout serial culture passage—UT7/Epo-S1 cell line (**A**) E2 (**B**) and B12 (**C**) clones were maintained in culture during 67 days after thawing with up to 21 culture passages. At indicated time points, B19V permissivity was evaluated by measuring levels of VP2 mRNA and normalized with β-actin mRNA by the 2^−^^ΔCt^ method. The results shown are the means + SD of 3 independent measurements.

E2 and B12 clone showed a decrease of percentage of S/G2/M cells from, respectively, 75.8% and 82 % at day 7, to 60.4 and 53.4% at day 80, suggesting a higher stability of E2 cell line. B19V permissivity stability was also analyzed throughout serial culture passage and compared to UT7/Epo-S1 reference (Figure 8).

While UT7/Epo-S1 and B12 clone showed a decrease of sensitivity to B19V, E2 clones’ permissivity seems to be stable at high culture passage.

Overall, our results identify two highly permissive UT7 clones, B12 and E2, and show that the S/G2/M phase is essential for B19V sensitivity.

## 4. Discussion

Most of the currently available approaches focus on the detection of B19V DNA, but there is a need for a suitable in vitro method for the direct quantification of virion infectivity, for use in assessing neutralizing antibodies, to evaluate viral inactivation assays or in antiviral research field. However, efforts to develop such methods have been hampered by the lack of suitable B19-sensitive cell lines in vitro. We describe here a new cell model with high sensitivity to B19V infection. As expected, hematopoietic cell lines of different origins were heterogeneous but, surprisingly, our results also demonstrate considerable variability among cell lines derived from the same patient, all named UT-7/Epo. This variability of B19V sensitivity may depend on erythroid stage, B19V entry receptor expression and/or the activation of specific signaling pathways [7]. Our findings highlight the need for tracking criteria to ensure the stability of the cell line used. As we show here that B19V sensitivity is linked to S/G2/M cell cycle status, we propose the use of cell cycle status to define the optimal cells for selection and as a keeper of clone stability. This study proposes an improved cellular model for the detection of B19V infectious units, with a sensitivity up to 35 times higher than previously achieved.

B19V has an extremely strong tropism for human erythroid progenitor cells. Since the discovery that B19V inhibits erythroid colony formation in bone marrow cultures by inducing the premature apoptosis of erythroid progenitor cells [42], numerous approaches and studies attempt to find a method of virus culture in vitro. Primary [43,44] or immortalized [36] CD36^+^ erythroid progenitor cells (EPC) derived from hematopoietic stem cells were the most permissive cell models for B19V infection. CD36^+^ EPCs reflect the natural etiologic B19V cell host, but the main problem with the use of this model is the difficulty in obtaining a continuously homogeneous cell line, with respect to differentiation stage, proliferation rate and metabolic activity. Moreover, the reagents and cytokines required for cell culture (SCF, Il-3, Il-6, Epo) preclude the use of CD36^+^ EPCs for routine B19V cell-based detection methods. To counteract this lack of suitability, cancer cell lines constitute a sound, practical, cost-effective alternative model, overcoming these difficulties. In recent years, many cancer cell lines have been tested, but only a few erythroid leukemic (KU812) [35] or mega-karyoblastoid cell lines (UT-7) [26] with erythroid characteristics support B19V replication. In our study, we chose also to investigate TF-1 permissivity. The TF-1 cell line is derived from the bone marrow aspirate of an erythroleukemic patient [45]. These cells display marked erythroid morphological and cytochemical features common to CD36^+^ EPCs, and the constitutive expression of globin genes highlights the commitment of the cells to the erythroid lineage [46]. Surprisingly, Gallinella et al. showed that TF-1 cells allow only B19V entry, with impaired viral genome replication and transcription, as shown by the presence of single-stranded DNA, and the absence of double-stranded DNA and RNA in B19V-infected TF-1 cells [47]. As previously described, no B19V RNA was detectable in the TF-1 cell line. The cellular factors involved in the transcriptional activation of the B19V promoter contribute to the restriction of permissiveness. Two factors, erythropoietin (Epo) and STAT-5, are key factors involved in B19V replication and transcription [48]. TF-1 cells express a truncated and mutated form of the Epo receptor [49], leading to impaired STAT-5 activation [34]. In the TF-1-ER cell line, stable ectopic expression of a full-length Epo receptor restores Epo-induced proliferation and STAT-5 activation. Here, despite Epo receptor signaling and STAT-5 activation, we found no evidence of B19V transcription, reflecting the involvement of unknown processes in the molecular mechanisms controlling B19V permissivity.

The first cell line reported to be permissive for B19 infection was an Epo-dependent subclone of UT-7, a mega-karyoblastoid cell line [32]. In 2006, Wong et al. published a comparative study of B19V sensitivity and permissivity in various cell lines [22]. They obtained evidence for the B19V infection of UT7/Epo and KU812Ep6 cells, although the percentage of B19V-positive cells was low (<1% immunofluorescent B19V^+^ cells). UT7/Epo-S1, a subclone of UT7/Epo obtained by limiting dilution and screening for B19V susceptibility [26], had the highest sensitivity, with approximately 15% of the cells staining positive for B19V [29]. Permissivity is restricted to a subset of cells, but the degree of viral DNA replication in these cells is similar to that in EPCs [50,51]. Since its characterization, the UT7/Epo-S1 cell line has been widely used to investigate the molecular mechanisms of B19V infection and to develop antiviral strategies against B19 [28]. We used UT7/Epo-S1 as a reference and compared the sensitivity of UT7/Epo cells from different laboratories. B19V permissivity seemed to be similar in the various UT7/Epo cells, but UT7/Epo-STI cells displayed levels of B19V gene expression almost 10 times higher than those in UT7/Epo-S1 cells. UT7/Epo-STI cells have been cultured with great care to ensure the preservation of their erythroid features, and they undergo erythroid differentiation following treatment with JQ1, a Bet-domain protein inhibitor [33] or TGF-β1 [52]. However attempts to characterize cell lines have been hampered by the heterogeneity of continually evolving multiple sub-clonal leukemic populations, as revealed at the cytogenetic level by the unstable karyotype documented at various time points for UT-7: at the admission of the patient to hospital (44 chromosomes, XY), at the first cell line characterization (92 ± 6 chromosomes, XXYY) [27], in subsequent publication (82 ± 4 chromosomes, XXYY [53] and in our own cell line in 2017 (72 ± 13 chr., XXYY; unpublished data). This karyotype heterogeneity highlights the presence of heterogeneous subclones within cell lines and might account for the variation of B19V sensitivity among UT-7 cell lines and clones.

The cell cycle is known to be crucial for erythroid differentiation, ensuring precise coordination of the critical differentiation process by Epo and erythroid-specific transcription factors [54,55]. We decided to select clones on the basis of cell cycle status. The FUCCI system represents a convenient approach to track cell cycle as its readability allows analysis of living cells at a single cell level [56]. By using clones with different cell cycle status, we demonstrated a strong correlation between S/G2/M cell cycle status and permissivity. A total RNA analysis, with a comparison of transcriptomes from highly permissive (group III) and less sensitive (group I & II) clones would shed more light on the molecular mechanisms involved in B19V infection. Along with karyotype, study of permissive versus restrictive clones could lead to the discovery of key molecular factors for B19V permissivity.

B19V has been shown to induce cell cycle arrest at G2 phase [26,57], but the importance of cell cycle status for B19V entry has not been investigated. A complex combination of multiple factors, including differentiation stage, specific cell cycle status, surface receptor and co-receptor, signaling pathways and transcription factors, may account for the difficulty of identifying the best cellular model for completion of the B19 viral cycle. We describe here two clones, E2 and B12, with a permissivity for B19 up to 35 times higher than that of the previously described references. By comparison with their less sensitive counterparts (groups I & II), these new highly permissive cell models (group III) constitute a potential advance towards understanding the crucial molecular determinants of B19V infectivity.

In addition to the use of E2 and B12 clones to investigate the molecular mechanisms of B19 infection, cell-based methods can be used for the detection/quantification of B19 infectious units, at low levels (<10^4^ DNA geq), in human fluids and tissues. There is a need for a practical in vitro method for the direct quantification of virion infectivity, as applied to the screening and/or assessment of neutralizing antibodies, antiviral drugs, and viral inactivation assays.

In the context of plasma-derived medicinal products, due to the lack of a suitable in vitro culture assay for B19, animal parvoviruses are currently used as a model for B19V, to assess B19 viral reduction during manufacturing processes. However, it remains unclear whether these models accurately reflect the behavior of B19V. Yunoki et al. reported an unexpected greater resistance of B19V than of canine parvoviruses (CPVs) to heating [58]. Mani et al. pointed up a discrepancy between animal and human parvovirus in a comparative study, describing the remarkable stability of the B19 viral genome in its encapsidated state [59]. Animal model parvoviruses display a certain resistance to heat inactivation [60,61] and pH stability [62], but comparative studies have indicated that they may behave differently from human B19 [2]. As E2 and B12 were the most sensitive cells in our study, with a permissivity up to 35 times higher than that of previously established references, they could allow the use of human parvovirus for the testing of viral inactivation processes, and the results of these tests would reflect the behavior of the native human virus.

Given the severity of B19V infection in immunocompromised patients, the development of antiviral strategies and drugs directed against B19V is of the highest relevance [28]. Depending on the immune state of the infected patient, acute infections can be clinically severe, and an impaired immune response can lead to persistent infections. The administration of high-dose intravenous immunoglobulins (IVIG) is currently considered the only available option for neutralizing the infectious virus [23,63]. In addition to the use of IVIG, the discovery of antiviral drugs with significant activity against B19 would offer important opportunities in the treatment and management of severe clinical manifestations. Two factors have critically limited the search for compounds to date. Firstly, the lack of a standardized and sensitive in vitro cell culture model has hampered advances in this field. Due to its usefulness, practicability and sensitivity, our cell model could replace the use of CD36^+^ EPCs and UT7/Epo-S1 cells in the discovery and evaluation of antiviral candidate compounds. Secondly, antiviral research requires native B19 infectious particles. However, B19V particles from viremic patients limit the feasibility of high-throughput screening against the available chemical libraries. No appropriate system for cell culture and in vitro virus production are available to date. UT-7 cells have been reported to produce infectious viral particles in vitro [43,64], but only a few UT-7 cells are infected and virions are produced in small numbers [65,66,67]. The strategies used here for the selection of clones permissive for B19V could also be used to select highly productive clones. Our most permissive clones, E2 and B12, could be challenged and assessed for in vitro viral production.

## 5. Conclusions

Altogether, we propose here an improved cell model with a high degree of permissivity to B19V, allowing the sensitive detection of infectious particles of B19. This finding opens up challenging new perspectives for basic research on the B19V life cycle. It may also offer opportunities for improving key steps in a number of critical applied approaches, including the sensitive evaluation of B19V virions in manufactured blood-derived products, and new strategies for B19V production in vitro.

## Supplementary Materials

The following are available online at https://www.mdpi.com/1999-4915/12/12/1467/s1, Figure S1: Cell cycle profile of the exponentially growing UT7/Epo-FUCCI cell line and clones, Figure S2: Comparison of relative levels of B19 mRNA and percentage of cells in respective cell cycle status. Figure S3: Cell cycle stability of E2 and B12 clones during serial culture passage.
