# Supplementary material for: Enhanced Cell-Based Detection of Parvovirus B19V Infectious Units According to Cell Cycle Status
Source: Viruses. 2020 Dec 18;12(12):1467. doi: 10.3390/v12121467 (PMC7766612; doi:10.3390/v12121467)
Supplement: Supplementary file 1 [file viruses-12-01467-s001.zip › viruses-896177-supplementary.pdf]

Enhanced cell-based detection of Parvovirus B19V infectious units according to cell cycle status.

Celine Ducloux, Bruno You, Amandine Langelé, Olivier Goupille, Emmanuel Payen, Stany Chrétien , Zahra Kadri

Supplementary Figures

Supplementary Figure 1

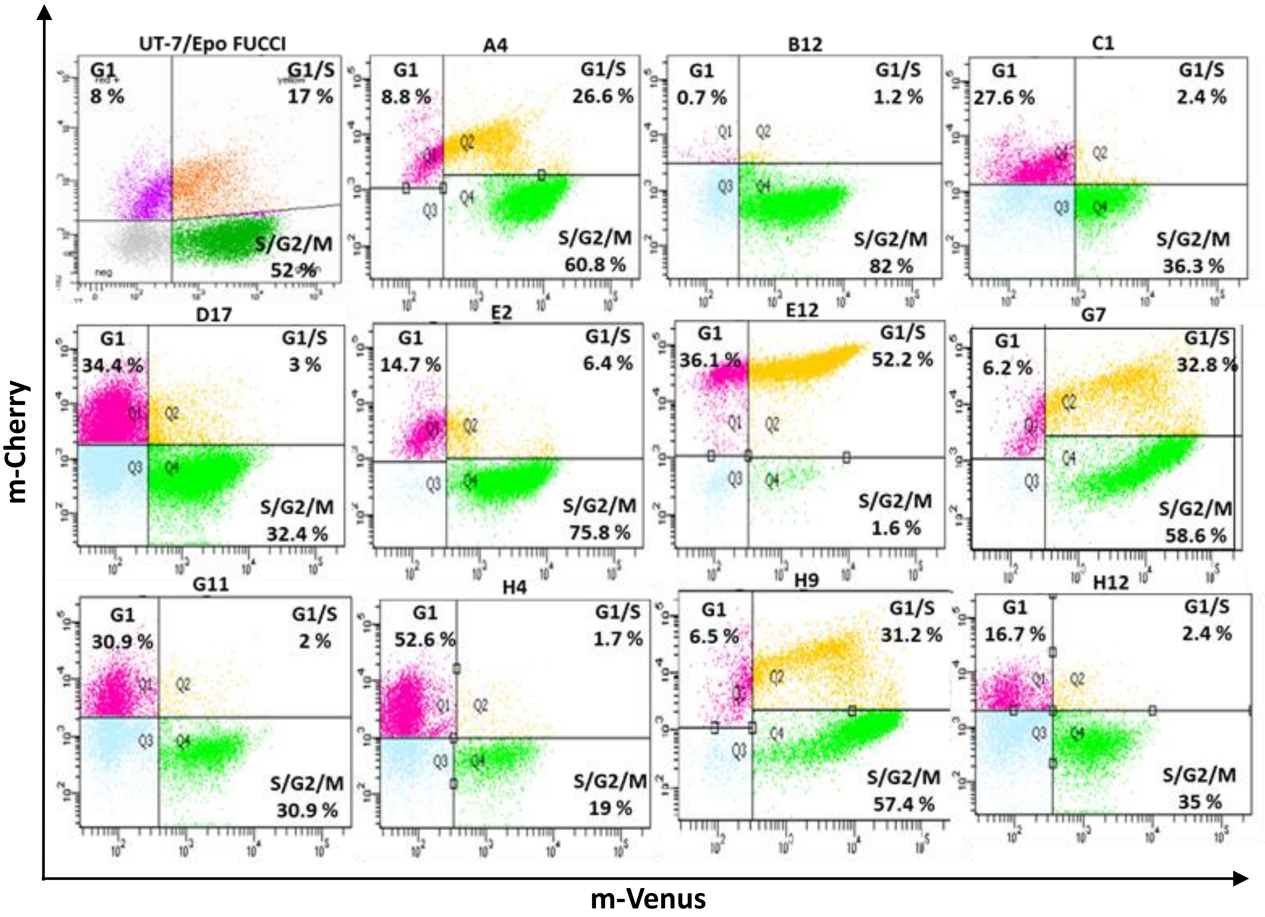

Figure S1 – Cell cycle profile of the exponentially growing UT7/Epo-FUCCI cell line and clones. The profile shown, as determined by flow cytometry (m-Venus on the *x*-axis; m-Cherry on the *y*-axis), corresponds to one representative experiment from four performed

Supplementary Figure 2

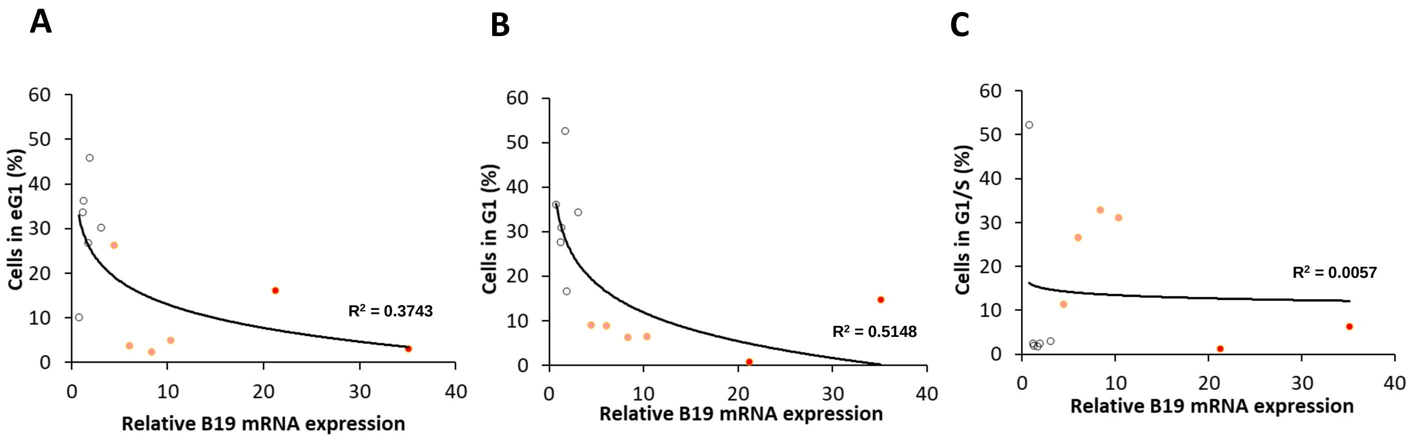

Figure S2 (related to Figure 6) - Comparison of relative levels of B19 mRNA and percentage of cells in respective cell cycle status. Percentage of cells in : A) early-G1 phase (e-G1), B) G1 phase, C) transition from G1 to S phase (G1/S) . Each dot correspond to the mean result for a single cell line or clone (*n*=3) assigned to groups I (white marks ○), II (orange marks ●) and III (red marks ●). A logarithmic transformation of the linear regression analysis and R^2 values are presented.

Supplementary Figure 3

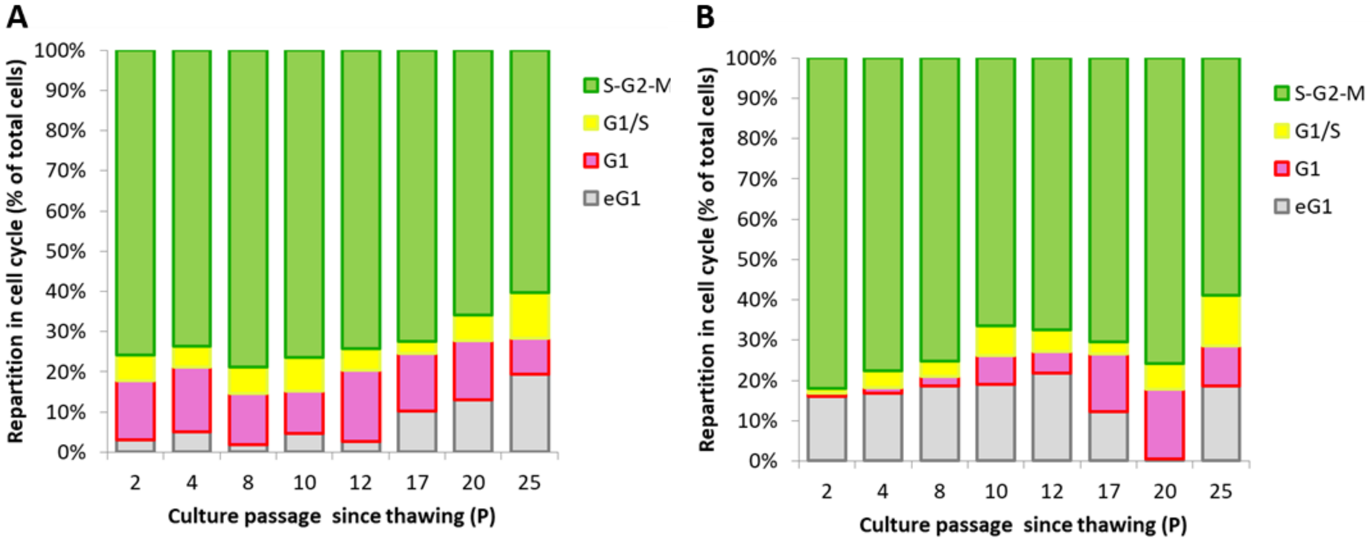

Figure S3 – Cell cycle stability of E2 and B12 clones during serial culture passage. UT7/Epo-FUCCI E2 A) and B12 B) clones were maintained in culture during 80 days after thawing with up to 25 culture passages. At indicated time points, cell cycle was evaluated by flow cytometry at exponential growth according to specific FUCCI protein expression. The results shown are the means of 3 independent measurements.
